# Bilateral Ovarian Agenesis and Bone Modeling Disease in Pre-puberty Girl With Primary Amenorrhea

**DOI:** 10.7759/cureus.75384

**Published:** 2024-12-09

**Authors:** Rami AlRbeihat, Ala Hindawi, Laith Qatarneh, Haneen Mahasneh, Jamil Marabha, Dana Ibrahim, Rana Hazeem, Farah Salman

**Affiliations:** 1 Department of Obstetrics and Gynecology, Royal Medical Services, Amman, JOR

**Keywords:** chromosome x translocation, hormonal replacement therapy, madelung deformity, ovarian agenesis, primary amenorrhea

## Abstract

Ovarian agenesis (OA) is a rare congenital condition characterized by the absence of one or both ovaries, often associated with chromosomal abnormalities, hormonal imbalances, and structural deformities. The condition is frequently diagnosed in females presenting with primary amenorrhea and delayed sexual development. This case report highlights a unique presentation of bilateral ovarian agenesis in a patient with chromosome X translocation, bone modeling disease, and primary amenorrhea.

A 17-year-old female with primary amenorrhea and a Madelung deformity presented with wrist pain, paresthesia, and limited range of motion. Imaging revealed delayed joint growth, a fragility fracture, and osteoporosis. Further evaluation uncovered a small uterus and absent ovaries on ultrasound and magnetic resonance imaging (MRI). Hormonal analysis showed elevated gonadotropins, follicle stimulation hormone and luteinizing hormone (FSH and LH), low estradiol, and low anti-mullerian hormone (AMH) levels. Laparoscopy confirmed rudimentary bilateral ovaries and chromosomal analysis revealed 46,X,der(X)t(X;3)(p11;p11), indicating an X chromosome translocation with an *SRY* gene microdeletion. The patient was diagnosed with bilateral ovarian agenesis and referred for multidisciplinary care.

Treatment included hormonal therapy with progyluton and estrofem, transitioning to marvilon, alongside physical therapy, nutritional support, and psychological counseling. After six months, the patient showed improvements in Tanner's score, weight, mood, and bone density (transition from osteoporosis to osteopenia). Menstruation was restored, reflecting the success of the combined hormonal therapy and supportive treatments.This case underscores the importance of integrating cytogenetic, hormonal, and clinical evaluations in diagnosing and managing rare presentations of ovarian agenesis. Early hormonal therapy and multidisciplinary care can significantly improve physical and psychological outcomes, including restoring menstruation and bone density. This is the first reported case of bilateral ovarian agenesis with chromosome X translocation presenting with phenotypic amenorrhea and bone deformities, demonstrating the value of tailored therapeutic approaches. Ongoing monitoring remains essential to ensure continued progress and mitigate long-term risks.

## Introduction

Ovarian agenesis (OA) is one of the rarest conditions in obstetrics and gynecology. OA is described in the literature as the absence of one or both ovaries, with unilateral absence being more common [1,2]. The precise incidence of OA remains unknown, but it is estimated to occur in approximately 1 in 11,240 cases [3]. Generally, OA could rise due to multiple causes like genetic factors such as chromosomal abnormalities that can be seen in Turner syndrome or genetic mutations on the X chromosome that may lead to OA, such as in our presented case [4,5]. Additionally, insufficient levels of sex hormones, whether it is testosterone or estrogen, may contribute to the underdevelopment of the ovaries [6].

Other hypotheses, like embryogenic factors, suggest that a defect in embryogenesis during gonadal ridge differentiation could lead to congenital agenesis of the ovaries. Such presentation can be seen in Mayer-Rokitansky-Küster-Hauser (MRKH) and androgen insensitivity syndromes [7,8]. Regarding environmental factors, there is a controversy in the literature on whether teratogenic exposure during pregnancy can cause embryogenic malformations of the female genital tract. Most studies suggest a minimal risk to the fetus from specific medications and radiation types [9,10]. While OA can present with many syndromes and conditions, it could still be due to idiopathic reasons in some cases.

Women with this condition typically present with primary amenorrhea, although some cases are diagnosed incidentally during laparoscopy or laparotomy for unrelated causes [2]. Most cases may present with a variety of congenital complications like webbed neck, malformations of the feet, cubitus valgus, and cervical spine abnormalities [11]. Furthermore, young OA patients are often of long stature, underweight, and present with primary amenorrhea, delayed development of sexual characteristics, and sexual hormone imbalances like progesterone, estrogen, and testosterone due to absence/insufficiency of ovaries [12,13]. Diagnostic imaging techniques such as ultrasound (US) and MRI play a crucial role in identifying OA. US imaging is the most commonly used technique, while 49% of reported cases are diagnosed laparoscopically [1]. Hormonal replacement therapy has been proven to be effective in females diagnosed with amenorrhea, with evidence of its influence on restoring menstruation, bone density, and weight gain [14,15].

In this article, we present a case of bilateral ovarian agenesis with bone modeling disease.

## Case presentation

**Figure 1:**
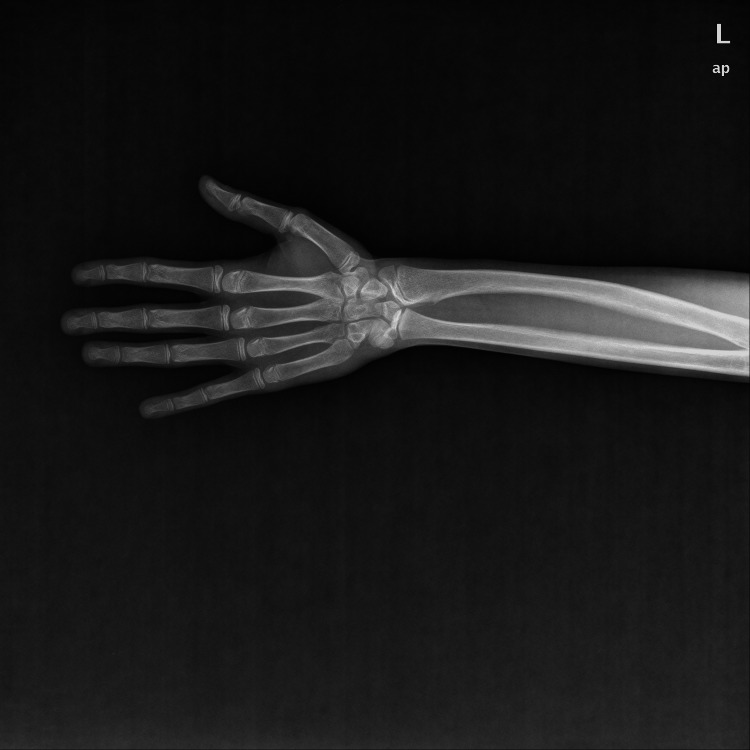
Left wrist X-ray showing bone modeling disease.

**Figure 2:**
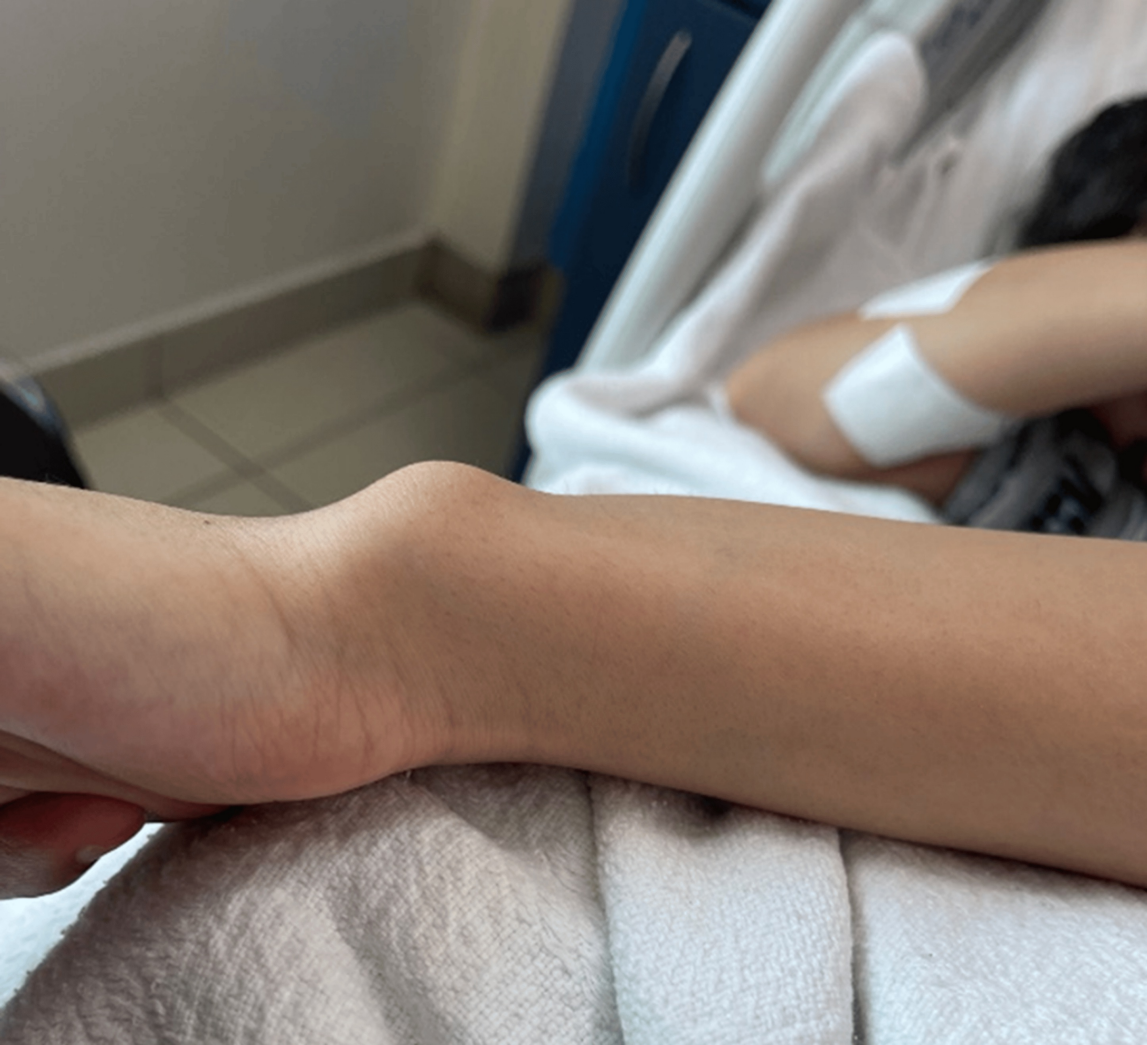
Left wrist physical examination showing bone modeling disease.

A 17-year-old female patient with a known case of Madelung deformity and primary amenorrhea presented with a variety of complications and disorders. Initially, the patient visited our orthopedic department complaining of wrist pain associated with limitation in range of motion (ROM), paresthesia and numbness, and bulging was noticed at the ulnar side of the wrist. X-ray was requested and showed evidence of delayed joint growth and a fragile fracture at the radius styloid process level (Figure 1, Figure 2).

The patient reported stomach pain with vomiting and diarrhea for four days. Physical examination revealed negative rebound, psoas, and Rovsing’s signs, with epigastric, right upper quadrant, and suprapubic tenderness that had worsened. The patient had recently visited the emergency room (ER), was admitted with medication, and had vital signs of BP (103/60 mmHg), HR (88 bpm), and temperature (36.7°C). Further investigations, including lab tests and abdominal X-rays, were normal, and endoscopy showed no issues. The patient was referred to gynecology as a known case of primary amenorrhea for further evaluation.

**Figure 3:**
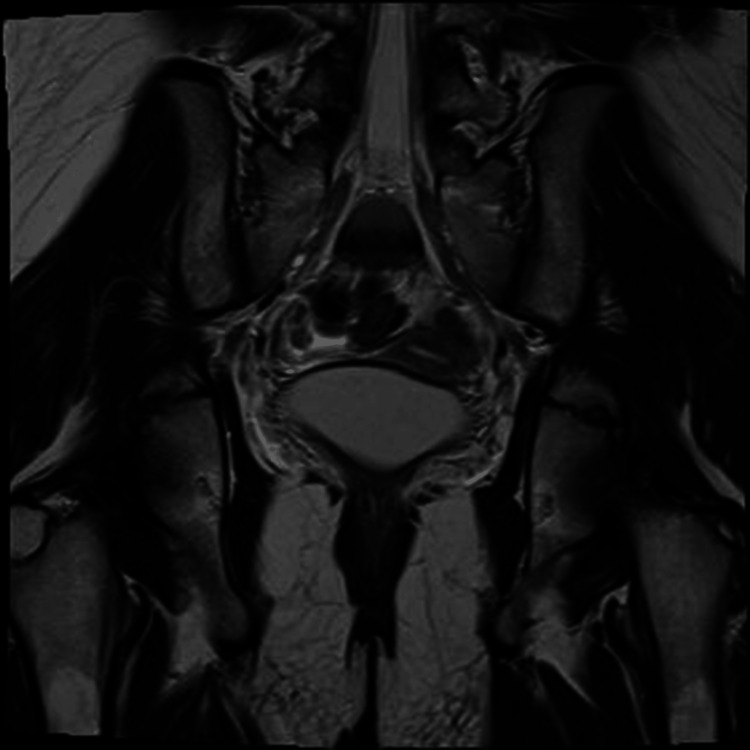
Magnetic resonance imaging (MRI) of the abdomen showing an absent uterus.

A female obstetrician/gynecologist examined this patient. She was 34 kg in weight and 161 cm in height. Tanner's score for breast development, pubic hair, and axillary hair were all stage 2, and genitalia and hymen were normal. A US scan reported a small size uterus while ovaries could not be properly visualized, which further required an MRI of the pelvic region. The MRI results confirmed the previous findings, showing uterine dimensions of 1.8×1.3×3.2 cm³. The ovaries were not clearly identifiable, likely due to atrophy or being too small to locate, and no free fluid was observed in the pelvis (Figure 3).

Laboratory tests for blood hormonal levels were ordered and showed high levels of follicle-stimulating hormone (FSH) and luteinizing Hormone (LH), normal levels of dehydroepiandrosterone sulfate (DHEAS), low levels of estradiol (E2), which is also an indicator for menopause or estrogen deficiency/underproduction, low levels of anti-mullerian hormone (AMH) indicating low egg count, and normal dihydrotestosterone (DHT) levels (Table 1).

**Table 1:** Laboratory hormonal findings. FSH: follicle stimulating hormone. LH: luteinizing hormone. DHEAS: dehydroepiandrosterone sulfate. E2: estradiol. DHT: dihydrotestosterone.

Test	Result	Reference range
FSH	98.56 (IU/L)	Women (pre-menopause): 3-20
LH	34.24 (IU/L)	Women (follicular phase): 1.9-12.5 Women (mid-cycle peak): 8.7-76.3
DHEAS	183.3 (µg/dL)	Women (20-50): 45-320
Estradiol (E2)	5 (pg/mL)	Women (pre-menopause): 30-400
Dihydrotestosterone (DHT)	279.9 (ng/dL)	Women: 24-368

**Figure 4:**
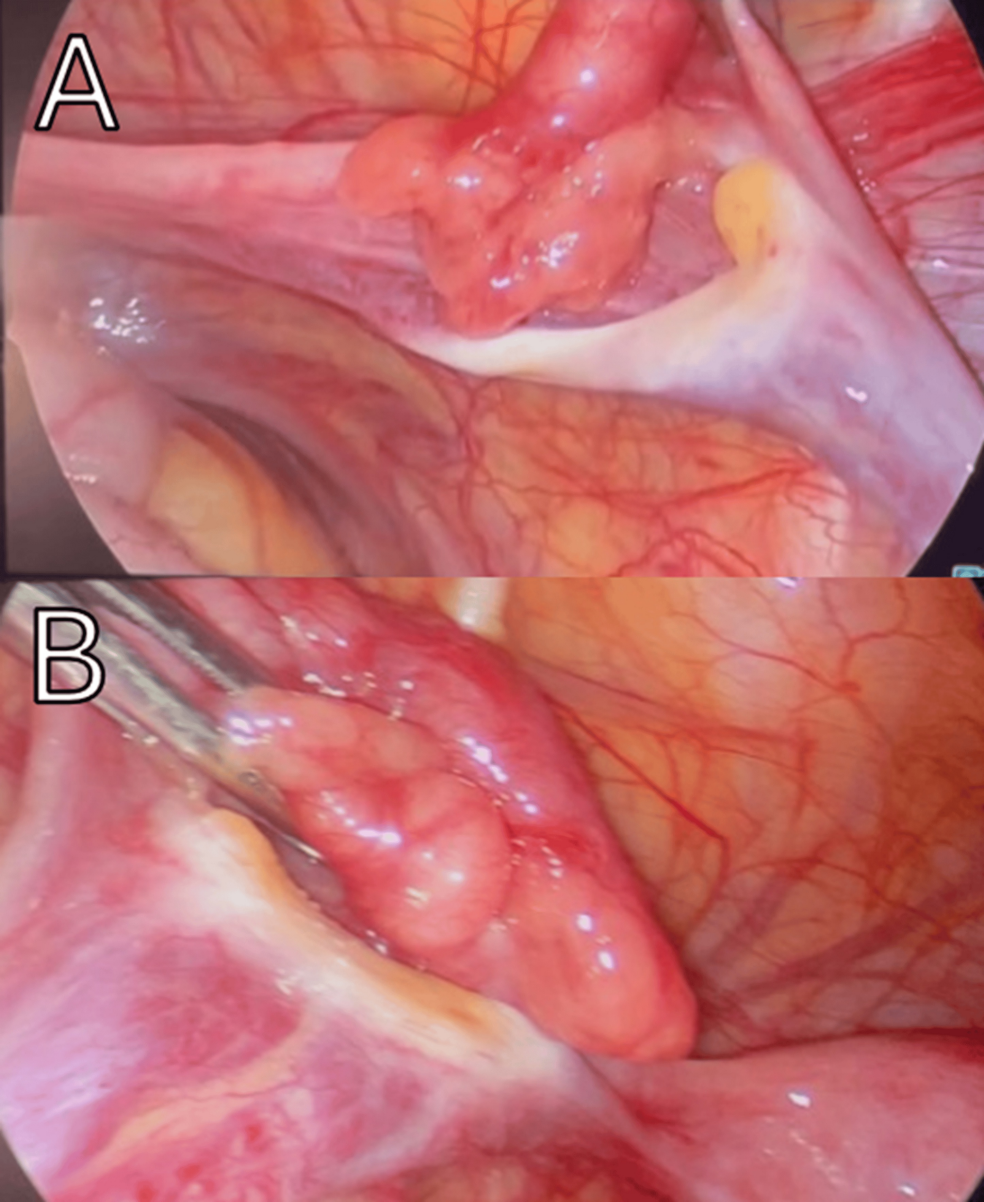
Intraoperative laparoscopic view of the abdomen. A) Absent right ovary B) Absent left ovary.

Based on these findings and poor imaging results, we decided to schedule a diagnostic operation. A laparoscopic approach was used, starting with the insertion of a uterine manipulator to help with visualization and movement of the pelvic organs. A 5mm incision was made at the umbilicus, and a Veress needle was inserted into the peritoneal cavity. Once the intra-abdominal pressure reached 15 mmHg, an optical trocar was placed through the umbilicus for direct visualization. Additional 5mm trocars were inserted on both sides of the lower abdomen to allow access for surgical instruments. This technique provided good access to perform the procedure with minimal disruption to surrounding tissue. Upon inspection, the uterus and both fallopian tubes appeared normal. However, both ovaries were noted to be scanty bilaterally, and the left ureter was slightly dilated (Figure 4). Hemostasis was secured, and the correct instrument count was verified three times. Surgery was uneventful, and the procedure was completed with appropriate closure, and dressing was applied.

Given the intraoperative findings of scanty ovaries and previous hormonal laboratory results, we ordered a chromosomal analysis, which showed a result of 46,X,der(X)t(X;3)(p11;p11), indicating an abnormality involving the X chromosome with a derivative translocation between chromosomes X and 3 at the p11 position. This suggests a structural chromosomal abnormality. The fluorescence in situ hybridization (FISH) analysis for the *SRY* gene (sex-determining region Y) revealed a microdeletion, confirming the absence of the *SRY* gene. This indicates that the *SRY* gene, responsible for initiating male sex determination, is missing.

**Figure 5:**
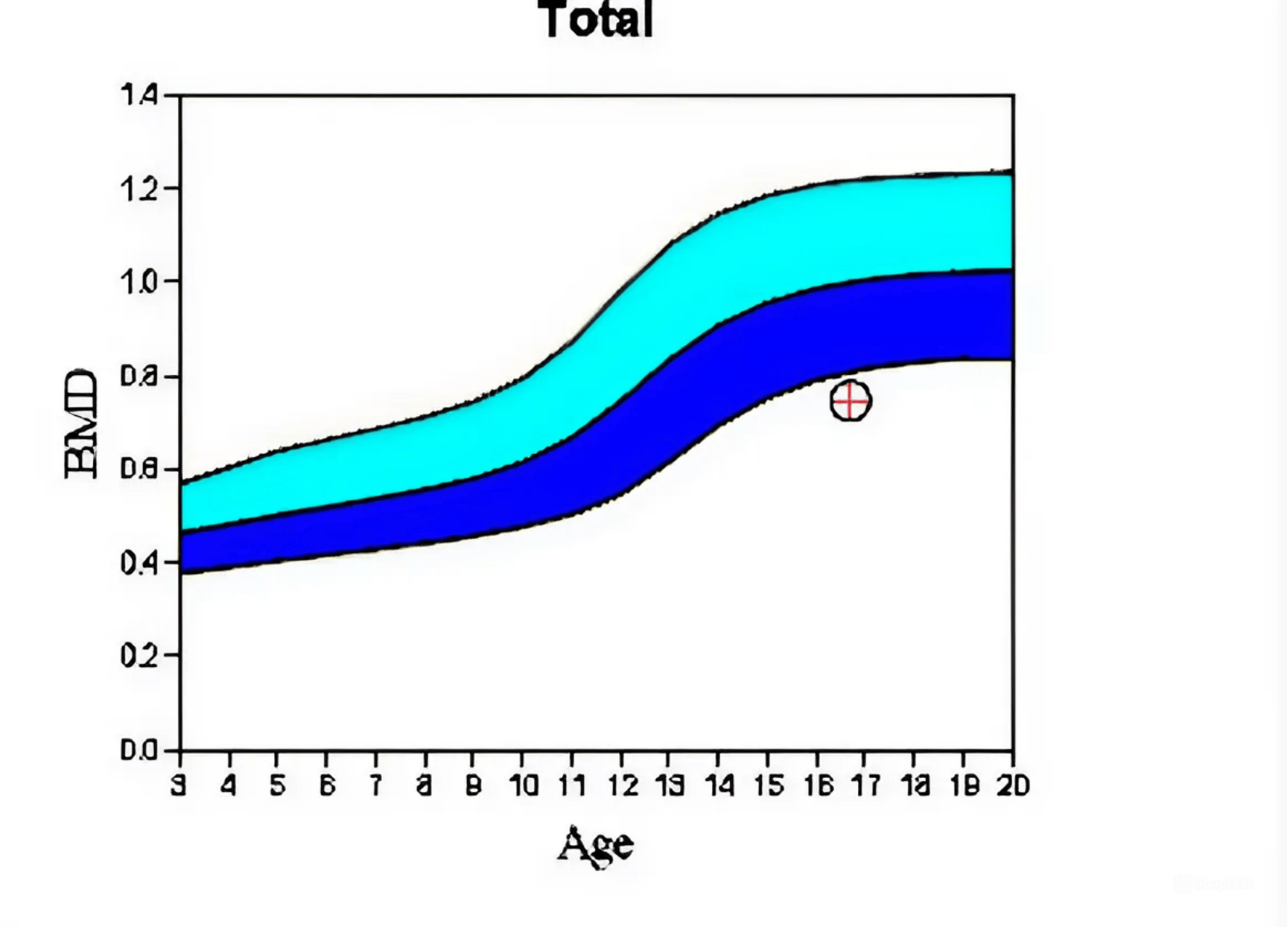
Bone scan (osteoporosis).

Given these genetic findings, combined with the clinical presentation, we suspect that the patient has bilateral ovarian agenesis, a condition often associated with abnormal chromosomal patterns like this one, which can disrupt normal ovarian development and function, possibly leading to low levels of estrogen which could directly influence growth hormones (GH), causing over-growth of the epiphyseal plates due to absence of inhibitors regulated by sexual hormones. Adding that the patient has a history of Madelung deformity, a bone modeling disorder, and an unhealed fracture, we requested DEXA scans to evaluate the bone density, and the results showed evidence of osteoporosis (Table 2, Figure 5).

**Table 2:** Bone density results summary. Area (cm²): The measured area of the region in square centimeters. BMC (g): bone mineral content in grams. BMD (g/cm²): bone mineral density in grams per square centimeter. T-score: standard deviations comparing BMD to a healthy young adult reference (not provided for individual vertebrae). PR (peak reference): percentage of peak bone mass (data unavailable). Z-score: standard deviations comparing BMD to age-matched norms. AM: age-matched percentile.

Region	Area (cm²)	BMC (g)	BMD (g/cm²)	T-score	PR (Peak reference)	Z-score	AM (age-matched)
L1	11.60	8.13	0.700	-	-	-	-
L2	10.86	8.11	0.747	-	-	-	-
L3	11.00	8.01	0.728	-	-	-	-
L4	13.06	10.47	0.802	-	-	-	-
Total	46.51	34.72	0.746	-	-	-2.6	75

**Figure 6:**
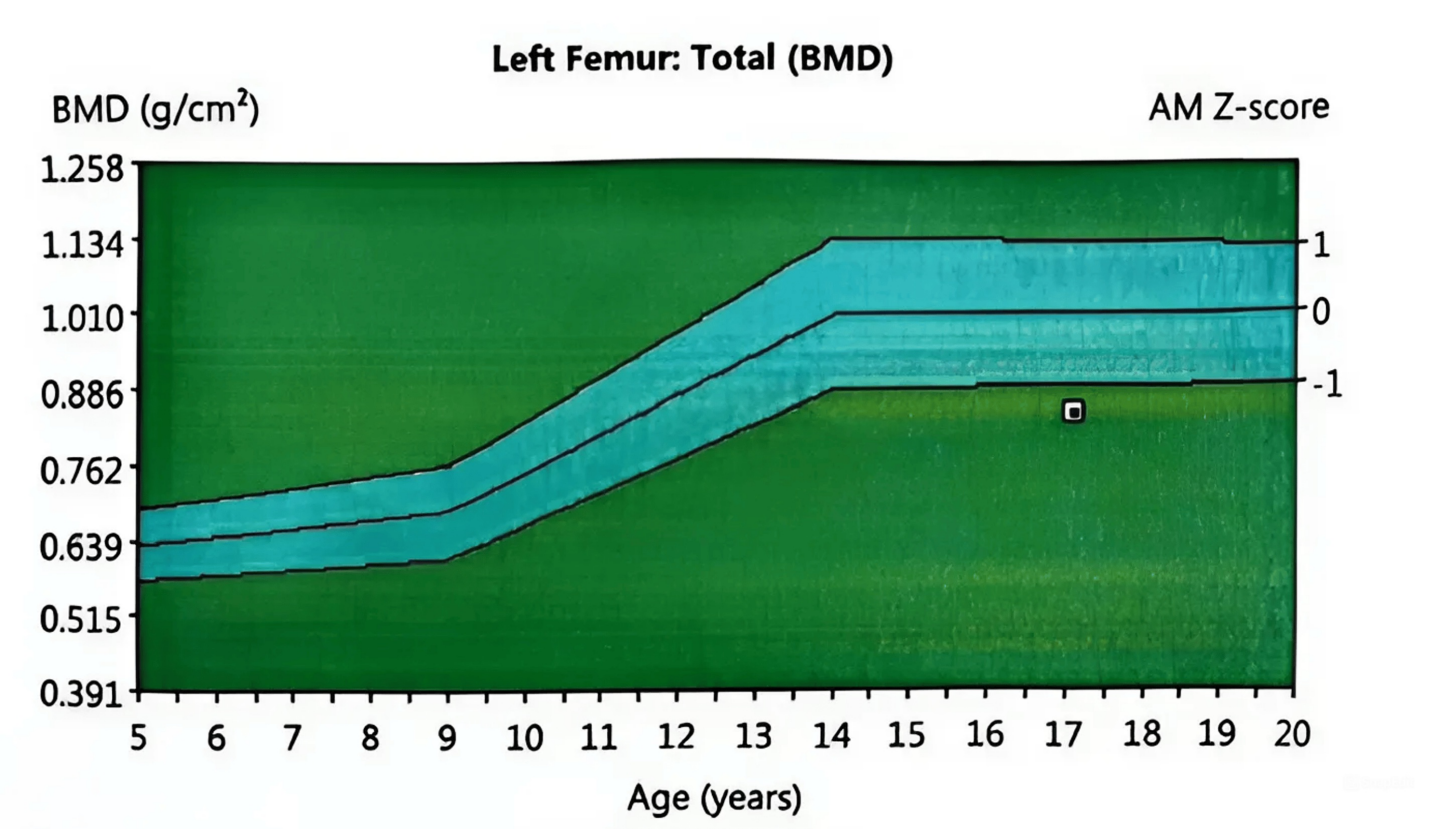
Bone scan (osteopenia). BMD (g/cm²): bone mineral density in grams per square centimeter.

We started the patient on estradiol valerate and norgestrel hormonal treatment for 6 months with a thin shift to ethinylestradiol, and we scheduled a follow-up visit for the patient every three months. Additionally, the patient was referred to the physical therapy clinic by a dietician and a psychologist. On the first visit, the patient scored 3 on the Tanner scale, showing signs of improvement in breast size, hair growth, and weight gain of four kg, with an improvement in mood. The results of a new DEXA scan showed signs of osteopenia, which indicates improvement and an increase in bone density compared to the old results of osteoporosis (Table 3, Figure 6). Moreover, the patient reported getting her first period. Our plan for this patient is to further re-evaluate hormonal levels, the state of the menstrual period, and bone density on the next appointment.

**Table 3:** Hip densitometry: USA (combined NHANES/lunar). BMD (g/cm²): bone mineral density in grams per square centimeter. YA (%): percentage of the young adult reference population (data unavailable in this table). YA T-score: standard deviations comparing the patient’s BMD to a healthy young adult population. YA Z-score: standard deviations comparing the patient’s BMD to an age-matched population. AM: age-matched percentile.

Region	BMD (g/cm²)	YA (%)	YA T-score	YA Z-score	AM
Neck	0.755	-	-	-1.8	-
Upper neck	0.612	-	-	-	-
Lower neck	0.837	-	-	-	-
Wards	0.704	-	-	-1.6	-
Troch	0.781	-	-	-0.1	-
Shaft	0.922	-	-	-	-
Total	0.851	-	-	-1.2	-

The following Figure 6 shows signs of osteopenia, which indicates improvement and an increase in bone density compared to the old osteoporosis results.

## Discussion

Ovarian agenesis is often associated with congenital and chromosomal anomalies and sexual dysfunction. However, it is often diagnosed at puberty when secondary sexual characteristics and function are delayed and affected. In females, primary amenorrhea raises concerns for anatomical dysgenesis in the female reproductive tract [16,17]. In this rare case report, a female patient presented to our clinic with deformity and primary amenorrhea, which led us to perform a gynecological consult to establish the cause of her amenorrhea.

Primary amenorrhea has several causes; one of the most common is ovarian dysfunction, which can be seen with estrogen deficiency and high gonadotropins (FSH and LH), as seen in this patient [18]. Further laparoscopic evaluation of this patient strongly suggested ovarian agenesis, and karyotyping analysis revealed a partial translocation between chromosomes X and 3. Previous reports of gonadal dysgenesis confirm a wide variation of chromosomal abnormalities with translocation of the X chromosome. Nakamura et al. reported a case of a 20-year-old female with gonadal dysgenesis confirmed with 46,X,der(X),(pteràq21::p21àpter), with phenotypically tall stature that was attributed to *SHOX* gene [19]. The association between chromosomal abnormalities of the X chromosome and gonadal dysgenesis remains elusive, with some suggesting meiotic pairing dysfunction causing gonadal dysgenesis rather than just a gene deletion [20]. Translocations of the X chromosome are very rare and exhibit phenotypic variations that differ from those seen in autosomal translocations [21]. Individuals with balanced chromosomal structural rearrangements, which involve no gain or loss of genetic material, are typically expected to appear phenotypically normal. However, they face an increased risk of infertility, spontaneous abortion, embryo failure, and birth defects due to the potential for cytogenetically unbalanced pregnancies [22].

A study by Huang et al. showed that only 10 cases of chromosome X translocations were identified in the literature, with phenotypical characteristics including amenorrhea, ovarian agenesis or hypoplasia, and uterus hypoplasia. Of them, one case involved a 16-year-old female with amenorrhea, normal sexual characteristics, infantile uterus (2.4x2.0x1.4 cm^3^), and absent ovaries. The karyotype revealed 46,X,t(X;22)(q25;q11.2), with a correlation between the breakpoints and low AMH levels [23]. Ovulatory disorders can have significant psychological consequences; a study in 1986 found that adult women with Turner syndrome often experience psychiatric difficulties and impaired self-esteem [24]. Thus, female patients with primary or secondary amenorrhea are in huge need of mental and emotional support from either family or caregivers [25].

## Conclusions

This is the first report of bilateral ovarian agenesis with chromosome X translocation presenting with phenotypic amenorrhea, bone deformities, and osteoporosis, where the patient's symptoms were successfully managed with treatment. This case highlights the significance of integrating cytogenetic, hormonal, and clinical evaluations in patients with multiple phenotypical abnormalities. Furthermore, the integration of hormonal therapy and supportive care has shown that early intervention can lead to significant improvements in physical health and quality of life. Ongoing monitoring of hormonal levels and bone density will be crucial to ensure continued progress and mitigate long-term health risks associated with her condition.

## References

[1] Chen HA, Vijayakumar P, Taylor-Giorlando M (2021). Unilateral ovarian agenesis: a systematic review of the literature and report of two cases. J Pediatr Adolesc Gynecol.

[2] Princi D (2017). Ipsilateral fallopian tube and ovary agenesis or absence? case report and review of the literature. J Gynecol Wom Health.

[3] Uckuyu A, Ozcimen EE, Sevinc Ciftci FC (2009). Unilateral congenital ovarian and partial tubal absence: report of four cases with review of the literature. Fertil Steril.

[4] Wade WG, Young RJ, Creery RDG (1956). A case of gonadal dysgenesis or the Ullrich-Turner syndrome with androgenic manifestations. Arch Dis Child.

[5] Di-Battista A, Moysés-Oliveira M, Melaragno MI (2020). Genetics of premature ovarian insufficiency and the association with X-autosome translocations. Reproduction.

[6] Devesa J, Caicedo D (2019). The role of growth hormone on ovarian functioning and ovarian angiogenesis. Front Endocrinol (Lausanne).

[7] (2024). Mayer-Rokitansky-Küster-Hauser syndrome. https://medlineplus.gov/genetics/condition/mayer-rokitansky-kuster-hauser-syndrome/.

[8] (2024). Androgen insensitivity syndrome: medlineplus genetics. https://medlineplus.gov/genetics/condition/androgen-insensitivity-syndrome/.

[9] Cassina M, Cagnoli GA, Zuccarello D, Di Gianantonio E, Clementi M (2017). Human teratogens and genetic phenocopies. Understanding pathogenesis through human genes mutation. Eur J Med Genet.

[10] Cunha GR, Taguchi O, Sugimura Y, Lawrence D, Mahmud F, Robboy SJ (1988). Absence of teratogenic effects of progesterone on the developing genital tract of the human female fetus. Hum Pathol.

[11] Cutler HC, Silver HK (1953). Syndrome of ovarian agenesis (congenitally aplastic ovaries): short stature, multiple congenital abnormalities and high urinary gonadotropins in a two year, eight month old female. Pediatrics.

[12] Gordan GS, Overstreet WE, Traut HF, Winch AG (1955). A syndrome of gonadal dysgenesis: a variety of ovarian agenesis with androgenic manifestations. J Clin Endocrinol Metab.

[13] Shamran SG, Hammood SA (2022). A review : The relationships between ovary disease and tumor marker. Al-Kufa Univ J Biol.

[14] Frank-Herrmann P, Strowitzki T (2013). Amenorrhea: what to think about? [Article in German]. J Clin Endoc and Metabol-Aust J Clin Endoc and Metabol.

[15] Stohr G (1946). A case of primary amenorrhea. J Clin Endocrinol Metab.

[16] Gasner A, Rehman A (2023). Primary Amenorrhea. StatPearls [Internet].

[17] Kapczuk K, Kędzia W (2021). Primary amenorrhea due to anatomical abnormalities of the reproductive tract: molecular insight. Int J Mol Sci.

[18] (2008). Current evaluation of amenorrhea. Fertil Steril.

[19] Nakamura Y, Suehiro Y, Sugino N, Sasaki K, Kato S (2001). A case of 46,X,der(X)(pter→q21::p21→pter) with gonadal dysgenesis, tall stature, and endometriosis. Fertil Steril.

[20] Barnes IC, Curtis D, Duncan SL (1988). A duplication/deficient X chromosome in a girl with mental retardation and dysmorphic features. J Med Genet.

[21] Gupta N, Goel H, Phadke SR (2006). Unbalanced X; autosome translocation. Indian J Pediatr.

[22] Verdoni A, Hu J, Surti U (2021). Reproductive outcomes in individuals with chromosomal reciprocal translocations. Genet Med.

[23] Huang N, Zhou J, Lu W (2023). Characteristics and clinical evaluation of X chromosome translocations. Mol Cytogenet.

[24] Downey J, Ehrhardt AA, Gruen R, Bell JJ, Morishima A (1989). Psychopathology and social functioning in women with Turner syndrome. J Nerv Ment Dis.

[25] Klein DA, Paradise SL, Reeder RM (2019). Amenorrhea: a systematic approach to diagnosis and management. Am Fam Physician.

